# Obesogenic high-fat diet heightens aerobic glycolysis through hyperactivation of oncogenic KRAS

**DOI:** 10.1186/s12964-019-0333-7

**Published:** 2019-02-28

**Authors:** Dan Wang, Yawei Bi, Lianghao Hu, Yongde Luo, Juntao Ji, Albert Z. Mao, Craig D. Logsdon, Ellen Li, James L. Abbruzzese, Zhaoshen Li, Vincent W. Yang, Weiqin Lu

**Affiliations:** 1 Division of Gastroenterology and Hepatology, Department of Medicine, Stony Brook of University School of Medicine, Stony Brook, New York, 11794 USA; 2 Department of Gastroenterology, Changhai Hospital, Shanghai, China; 3 School of Pharmaceutical Science, Wenzhou Medical University, Wenzhou, Zhejiang China; 4 Centeer BioTherapeutics Ltd Co, Houston, TX USA; 5 Department of Cancer Biology, University of Texas MD Anderson Cancer Center, Houston, TX 77030 USA; 6 Division of Medical Oncology, Department of Medicine, Duke Cancer Institute, Duke University, Durham, North Carolina USA

**Keywords:** KRAS, Pancreatic cancer, Glycolysis, High-fat diet, Obesity, COX-2, Hexokinase 2, Lactate dehydrogenase

## Abstract

Oncogenic KRAS plays a vital role in controlling tumor metabolism by enhancing aerobic glycolysis. Obesity driven by chronic consumption of high-fat diet (HFD) is a major risk factor for oncogenic KRAS-mediated pancreatic ductal adenocarcinoma (PDAC). However, the role of HFD in KRAS-mediated metabolic reprogramming has been obscure. Here, by using genetically engineered mouse models expressing an endogenous level of KRAS^G12D^ in pancreatic acinar cells, we demonstrate that hyperactivation of KRAS^G12D^ by obesogenic HFD, as compared to carbohydrate-rich diet, is responsible for enhanced aerobic glycolysis that associates with critical pathogenic responses in the path towards PDAC. Ablation of Cox-2 attenuates KRAS hyperactivation leading to the reversal of both aggravated aerobic glycolysis and high-grade dysplasia under HFD challenge. Our data highlight a pivotal role of the cooperative interaction between obesity-ensuing HFD and oncogenic KRAS in driving the heightened aerobic glycolysis during pancreatic tumorigenesis and suggest that in addition to directly targeting KRAS and aerobic glycolysis pathway, strategies to target the upstream of KRAS hyperactivation may bear important therapeutic value.

## Background

Aberrant cell metabolism, such as the complex reprogramming of glucose, glutamine, and tricarboxylic acid (TCA) cycle, has been recognized as a hallmark of cancer [1, 2]. Metabolic alterations allow cancer cells to sustain higher proliferation rates, to survive in the adverse tumor microenvironment, to gain invasive and metastatic abilities, and to resist drug treatment [2–4]. Among diverse metabolic alterations, cancer cells exhibit dramatically increased glucose uptake and lactate production even in the presence of oxygen and functional mitochondria, known as aerobic glycolysis. As a major fuel, glucose not only can be metabolized through the conventional anaerobic glycolysis but also can branch out to the pentose phosphate pathway for the syntheses of ribonucleotides and the reducing equivalent NADPH. NADPH is required for the reductive biosynthesis of fatty acid and the scavenging of reactive oxygen species (ROS) [5, 6]. In addition, glucose can be converted to glycerol or serine for lipids or amino acids biosynthesis. Thus, by providing building blocks and reducing equivalents, glucose can partly fulfill the diverse requirements of cancer cells through the rearrangement of the established metabolic pathways towards the enhancement of aerobic glycolysis [5, 7, 8].

The genetic landscape of pancreatic ductal adenocarcinoma (PDAC) shows nearly ubiquitous mutations of *KRAS,* which are required for the initiation and progression of pancreatic ductal adenocarcinoma (PDAC) [6, 9]. Oncogenic KRAS is a master regulator of pancreatic cancer metabolism, and mutant Kras copy number can define metabolic reprogramming and therapeutic susceptibilities [7, 9, 10]. A critical function of oncogenic KRAS is to drive metabolic reprogramming towards aerobic glycolysis, which is achieved in part through transcriptional up-regulation of multiple key rate-limiting glycolytic enzymes, including hexokinase 2 (HK II) and lactate dehydrogenase A (LDHA) [3, 7, 8]. However, previous metabolic studies either employed oncogenic *KRAS* overexpression models [7, 8] or utilized endogenous levels of mutant KRAS for in vitro metabolic studies [10]. Thus, it is not clear whether oncogenic KRAS at an endogenous level that imitates a physiological condition is the sole driver for aerobic glycolysis in vivo*.*

Glucose from the carbohydrate-rich diet (CD) is expected to be a major source to fuel aerobic glycolysis [7, 8]. On the other hand, obesity driven by chronic consumption of high-fat diet (HFD) is found to be an important modifiable risk factor for PDAC. Importantly, obesogenic HFD promotes oncogenic KRAS-mediated PDAC with high penetrance. However, it is unknown whether HFD will have any impact on oncogenic KRAS-driven glycolysis. In this study, we utilized a mouse model with an endogenous level of *KRAS*^*G12D/+*^ to present evidence that obesogenic HFD synergies with oncogenic KRAS to promote aerobic glycolysis, leading to critical pathogenic responses in the path toward PDAC.

## Methods

### Genetically engineered mouse models

*Kras*^*LSL-G12D/+*^ mice, which possess the conditional knock-in of mutant *Kras*^*G12D*^, were obtained as described [11]. *fElas*^*CreERT*^ mice, which express tamoxifen-regulated Cre recombinase under full-length *Elastase* promoter specifically in pancreatic acinar cells, were developed as previously described [12]. Upon TM treatment, nearly 100% of pancreatic acinar cells express Cre recombinase [12]. *Kras*^*LSL-G12D/+*^ mice and *fElas*^*CreERT*^ mice were cross-bred to generate *fElas*^*CreERT*^*;Kras*^*LSL-G12D/+*^ double-transgenic mice (called *fElas*^*CreERT*^*;Kras*^*G12D/+*^ after TM) for targeted expression of *Kras*^*G12D/+*^ in pancreatic acinar cells. In addition, *Cox-2*^*flox/flox*^ mice were crossed with *fElas*^*CreERT*^*;Kras*^*G12D/+*^ mice to generate *Kras*^*LSL-G12D/+*^*;Cox-2*^*flox/flox*^*;fElas*^*CreERT*^ mouse model (called *Kras*^*G12D/+*^*;Cox-2*^*−/−*^ after TM). All animal experiments were reviewed and approved by the Stony Brook University Institutional Animal Care and Use Committee (IACUC).

### Animal treatment

f*Elas*^*CreERT*^, *fElas*^*CreERT*^*;Kras*^*LSL-G12D/+*^*,* and *Kras*^*LSL-G12D/+*^*;Cox-2*^*flox/flox*^*;fElas*^*CreERT*^ mice were given TM by peritoneal injection for 5 days to fully activate Cre recombinase in pancreatic acinar cells when the mice were 70 days old. According to the treatment plan, *Kras*^*G12D/+*^ and *fElas*^*CreERT*^ mice were fed with either carbohydrate-rich diet diet (CD, 71.8% carbohydrate energy intake, Test Diet DIO 58Y2) or an isocaloric high-fat diet (HFD, 60% fat energy intake, Test Diet DIO 58Y1 van Heek Series; Lab Supply, Fort Worth, TX). *Kras*^*G12D/+*^*;Cox-2*^*−/−*^ mice were fed with HFD. After 10 weeks of treatment, mice were euthanized, and the pancreata were harvested for further experiments.

### Immunohistochemistry

Immunohistochemical (IHC) staining was performed on pancreatic sections. Briefly, pancreata were fixed overnight in 4% paraformaldehyde and embedded in paraffin. Paraffin-embedded tissues were cut into 5 μm-thick sections. After deparaffinization and rehydration, tissue sections were subjected to antigen retrieval and then treated with 0.5% H_2_O_2_ to block the endogenous peroxidase. The treated sections were then incubated with primary antibodies against p-ERK (1: 200, Cat #sc-136,521, Santa Cruz, Dallas, TX, USA) at 4 °C overnight. After washing, the sections were incubated with the appropriate biotinylated secondary antibodies (Vector Laboratories, CA, USA) for 1 h, washed again in PBS, incubated with ABC reagent (Vector Laboratories, CA, USA) for 30 min, and then reacted with diaminobenzidine (DAB, Vector Laboratories, CA, USA). Sections were viewed on an Olympus IX70 microscope. The resulting sections were then counterstained with hematoxylin. Fiji ImageJ software was used to obtain data for quantification and statistical analyses.

### Quantification of Alcian blue staining

Pancreatic tissues were fixed, embedded in paraffin, and sectioned. Alcian blue staining was performed to evaluate PanIN lesions as described [13]. Briefly, pancreatic tissue slides were hydrated in distilled water and processed with 3% acetic acid for 3 min, followed by incubating with Alcian blue solution (Sigma-Aldrich, Louis, MO, USA) for 30 min at room temperature. The slides were then washed in running water for 2 min and subjected to nuclear-fast red for 1 min. To quantify the relative Alcian blue-positive areas, five random, non-overlapping images were obtained at a magnification of × 100. For each image, the Alcian blue-positive area and the total pancreatic area were scanned using Fiji ImageJ and the percentage of the Alcian blue-positive area was calculated.

### Protein isolation and Western blot analysis

Snap-frozen tissues were homogenized in 0.5–1 ml ice-cold lysis buffer (Millipore, MA, USA) with protease inhibitor cocktail tablets (Roche, Germany). Tissue homogenates were centrifuged at 15,000 g for 15 min at 4 °C, and the supernatant was collected. Protein lysate from tissue was aliquoted to determine protein concentration using a protein assay dye reagent concentrate (Bio-Rad, CA, USA). The lysates were separated by SDS-PAGE and then transferred to nitrocellulose membranes. The membranes were rinsed with PBS containing 0.05% Tween 20 (PBS-T) and probed with antibodies against HKII (1:500; Cat #sc-130,358, Santa Cruz Biotechnology Inc. Dallas, TX, USA), LDHA (1:1000; Cat #2012S, Cell Signaling Technology, Danvers, MA, USA) and β-actin (1:5000, Cat #A5316, Sigma-Aldrich, Louis, MO, USA). The membranes were then washed with PBS-T and probed with the respective secondary antibodies conjugated to horseradish peroxidase for 1 h at room temperature. Autoradiography or the Odyssey Imaging System (LiCor Biosciences, Lincoln, NE) was used to visualize protein bands. Stripping buffer (Thermo, MA, USA) was used for sequential blotting and reprobing with other antibodies to provide a loading control. ImageJ densitometry software was used to quantify individual bands.

### Ras activity assay

Levels of GTP-occupied Ras from mouse pancreatic lysates were measured using a Raf pull-down assay kit (Millipore, MA, USA) as previously described [14]. Briefly, snap-frozen pancreatic tissues were homogenized on ice in lysis buffer. Cellular debris was removed by centrifuging at 15,000 g for 20 min at 4 °C. Protein concentrations were measured. About 1000 μg of lysate were incubated for 50 min at 4 °C with agarose beads coated with Raf-Ras binding domain, and the beads were washed for 3 times with washing buffer. Active Ras was analyzed by immunoblotting with an anti-Ras primary antibody (1:2000, Cat #05–516, Millipore, MA, USA) using β-actin as sample loading control. The intensity of the bands generated from Western blot assay was quantified using ImageJ software, and the fold changes relative to Ras activity from *fElas*^*CreERT*^ mice were calculated.

### Statistical analysis

Comparison between two groups was analyzed by Student’s *t*-test unless otherwise indicated. A *p* value of less than 0.05 was considered to be statistically significant. Results are expressed as group mean ± SEM or mean ± SD. We used GraphPad Prism 6.0 for data analysis.

## Results

### High-fat diet enhances oncogenic KRAS-induced aerobic glycolysis compared to carbohydrate-rich diet

**Fig. 1:**
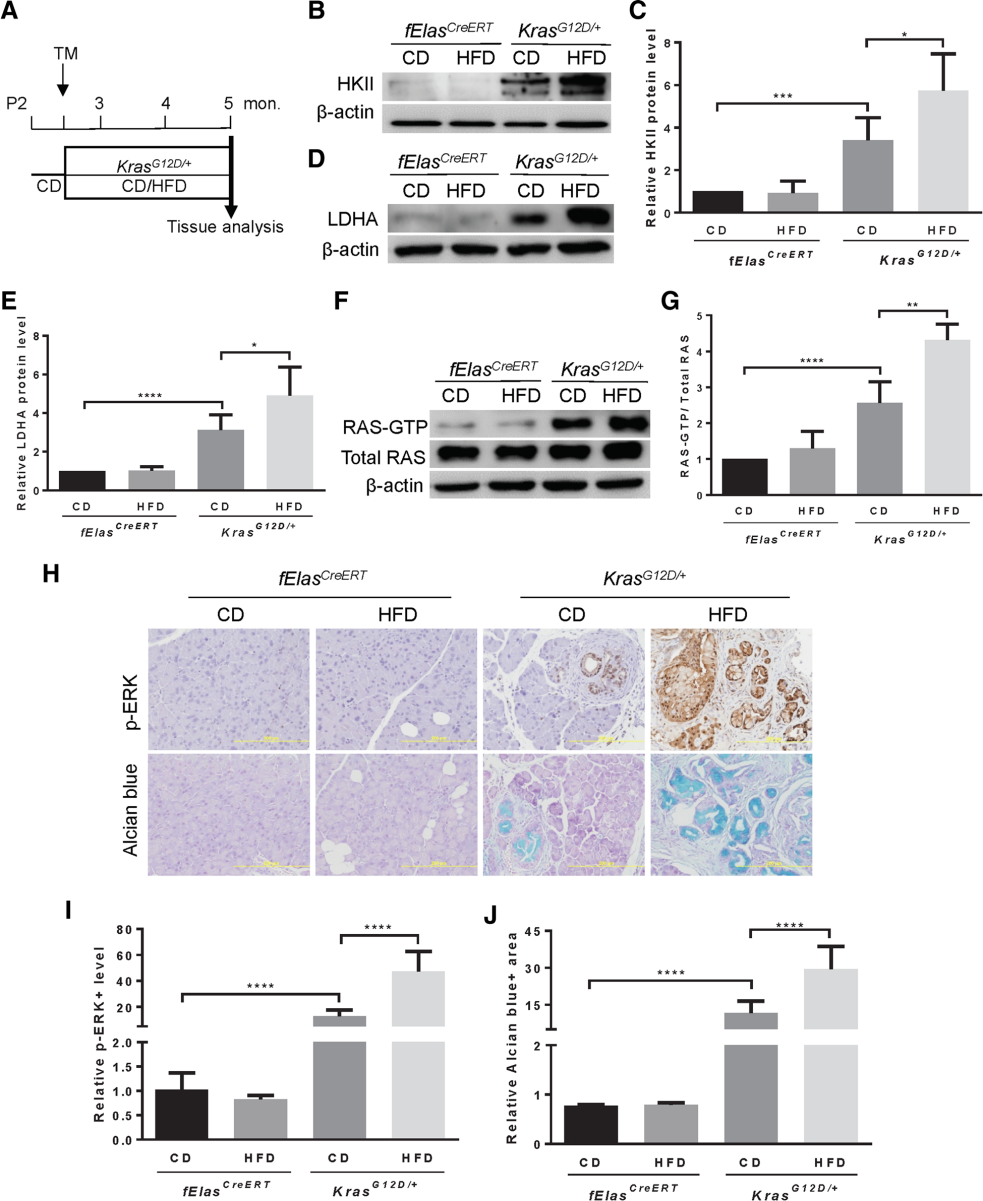
HFD hyperactivates oncogenic KRAS to elevate aerobic glycolysis. **a** Diagram of mouse treatment. TM, tamoxifen; CD (*n* = 8), carbohydrate-rich diet; HFD (n = 8), high-fat diet. **b-c** Western blot analysis and quantification of the expression levels of HKII in *fElas*^*CreERT*^ and *Kras*^*G12D/+*^ with the indicated treatment. Three independent experiments from three different mice per group were performed. **d-e** Western blot analysis and quantification of the expression levels of LDHA in *fElas*^*CreERT*^ and *Kras*^*G12D/+*^ with the indicated treatment. Three independent experiments from three different mice per group were performed. **f-g** RAS activity assay followed by Western blot analysis and quantification of the RAS-GTP levels in mice harboring *fElas*^*CreERT*^ and *Kras*^*G12D/+*^ under both CD and HFD for 10 weeks. Total RAS was used as a loading control. Three independent experiments from three different mice per group were performed. **h** Representative IHC staining for p-ERK (upper panel) and Alcian blue staining for acidic mucins (lower panel) as indicated. Magnifications: 200x. (*n* = 6 mice per group). **i** Statistical analysis of p-ERK (n = 6) as represented in Fig. 1h (upper panel). **j** Statistical analysis of the Alcian Blue staining (n = 6) as represented in Fig. 1h (lower panel). Data are mean ± SD. *p* values are determined by the Student’s *t*-test. *p < 0.05; **p < 0.01; ***p < 0.001; ****p < 0.0001

Evidence supports that acinar cells are the cell of origin for PDAC [13]. To determine the role of HFD in oncogenic KRAS-mediated aerobic glycolysis in cancer, we employed the *Kras*^*LSL-G12D/+*^*;fElas*^*CreERT*^ mouse model that expresses mutant *Kras*^*G12D*^ in one allele at an endogenous level in nearly 100% acinar cells upon tamoxifen induction (hereafter called *Kras*^*G12D/+*^) [11, 12, 15]. These mice were exposed to either a CD containing carbohydrates accounting for 71.8% of energy intake or an isocaloric HFD containing saturated fat for 61.6% of energy intake for ten weeks (Fig. 1a). The pancreata of both CD- and HFD-fed *fElas*^*CreERT*^ control mice with wild-type KRAS exhibited no significant differences in the levels of HKII, a key rate-limiting enzyme that catalyzes the phosphorylation of glucose, and LDHA that converts pyruvate to lactate in the aerobic glycolytic pathway (Fig. 1b-e). These data reveal that regardless of the nature of the diets, in the absence of oncogenic KRAS, levels of these two enzymes remained constant, indicating that the CD and HFD diets play no independent role in promoting aerobic glycolysis. As expected, the CD-fed *Kras*^*G12D/+*^ mice showed marked increases in both HKII (Fig. 1b-c) and LDHA (Fig. 1d-e) compared to the *fElas*^*CreERT*^ mice, indicating that oncogenic KRAS at an endogenous level promotes aerobic glycolysis. However, to our surprise, in the HFD-fed *Kras*^*G12D/+*^ mice, both HKII (Fig. 1b-c) and LDHA (Fig. 1d-e) were further increased significantly. These data suggest that HFD cooperates with KRAS^G12D^ to enhance aerobic glycolysis to a significantly higher level than CD, in which carbohydrate should be a more convenient and direct source to feed into glycolysis.

### Oncogenic KRAS hyperactivation underlies the heightened aerobic glycolysis under obesogenic HFD challenge

Oncogenic KRAS was viewed as being locked in a constitutively active state for the past thirty years [16]. However, recent studies have demonstrated that oncogenic KRAS, at an endogenous level, is not constitutively active and is therefore insufficient to induce PDAC [14, 17]. Rather, it requires to be further activated by stimuli, such as the chronic consumption of HFD, to drive PDAC [14, 15]. To determine whether the elevated glycolysis by HFD over CD corresponds to the activation state of RAS, we measured the changes in RAS activity. Results showed that the pancreata of the HFD-fed *Kras*^*G12D/+*^ mice exhibited significantly higher levels of GTP-bound RAS than CD-fed *Kras*^*G12D/+*^ mice (Fig. 1f-g). Analysis of mitogen-activated protein kinase (MAPK) pathway as the downstream effector of KRAS important for the remodeling of glucose metabolism revealed that the p-ERK level was markedly elevated in the HFD-fed *Kras*^*G12D/+*^ mice compared to CD-fed *Kras*^*G12D/+*^ mice (Fig. 1h, upper panel, and i). These data suggest that the HFD-promoted glycolysis is associated with a hyperactivation state of oncogenic KRAS. Consistent with the elevated glycolysis and KRAS hyperactivation, histological analysis revealed that acidic mucins, which mark metaplastic ductal cells in pancreatic intraepithelial neoplasia (PanIN) lesions, were substantially increased in the pancreata of HFD-fed *Kras*^*G12D/+*^ mice (Fig. 1h, lower panel, and j), which were in marked contrast to sporadic PanIN lesions in CD-fed *Kras*^*G12D/+*^ mice. All *fElas*^*CreERT*^ control mice were p-ERK negative and PanIN-free regardless of the nature of diets (Fig. 1h-j). These data suggest a rapid and wide-spread progression to PanIN lesions in accordance with KRAS^G12D^ hyperactivation and the elevation of aerobic glycolysis in the *Kras*^*G12D/+*^ mice under the challenge of obesogenic HFD rather than CD.

### Suppression of oncogenic KRAS hyperactivation attenuates aerobic glycolysis and inhibits PanIN lesions under chronic HFD challenge

**Fig. 2:**
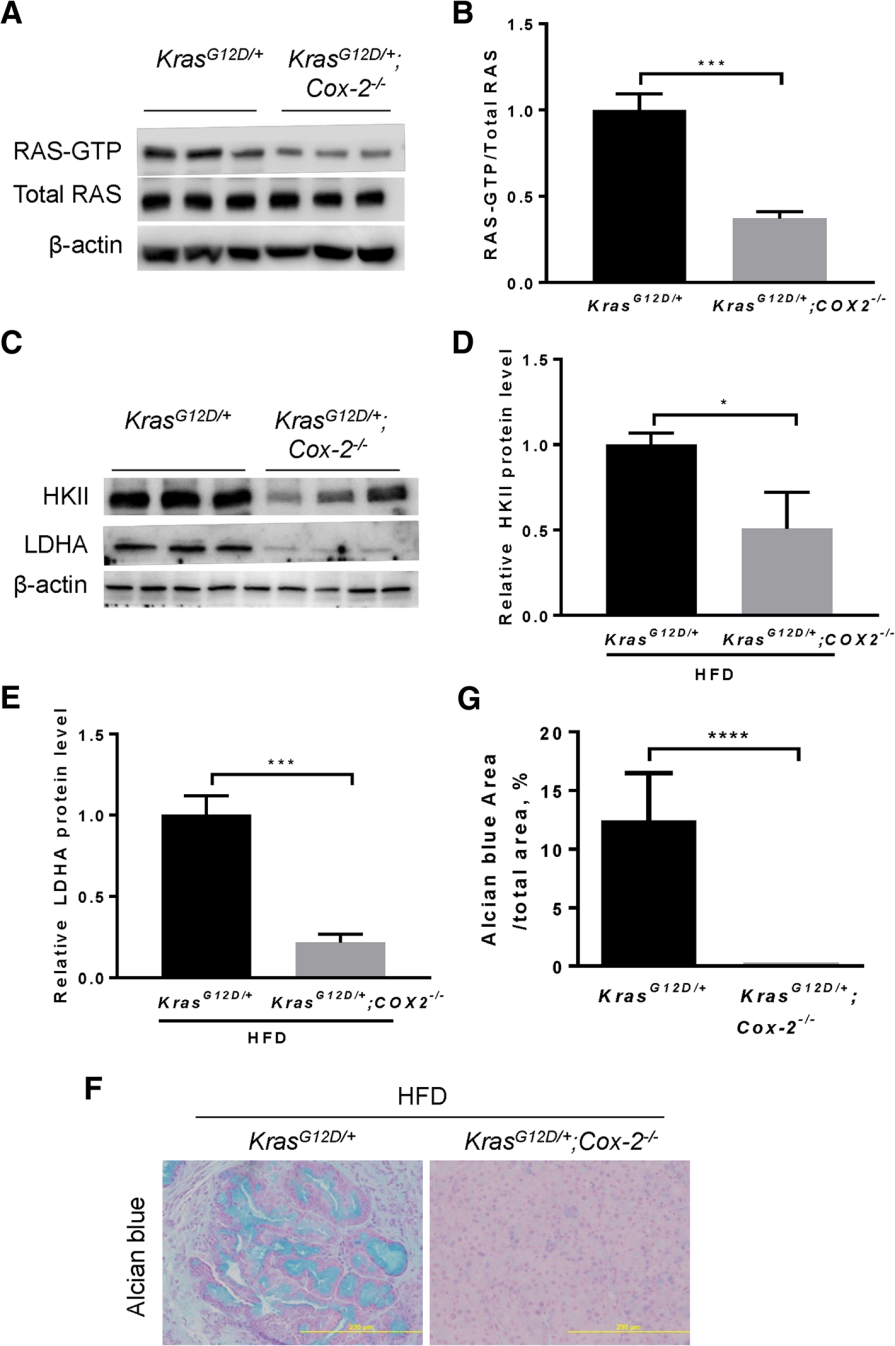
Cox-2 ablation suppresses oncogenic KRAS hyperactivation and aerobic glycolysis under obesogenic HFD challenge. **a-b** RAS activity assay followed by Western blot analysis and quantification of the RAS-GTP level in mice (*n* = 3 mice per group) harboring *Kras*^*G12D/+*^ and *Kras*^*G12D/+*^*;Cox-2*^*−/−*^ under HFD conditions. Total RAS served as a loading control. **c-e** Western blot and quantitative analyses of the expression levels of HKII and LDHA in mice (n = 3 mice per group) harboring *Kras*^*G12D/+*^ and *Kras*^*G12D/+*^*;Cox-2*^*−/−*^ under HFD conditions. **f-g** Representative Alcian blue staining and quantitative analyses for acidic mucins in the *Kras*^*G12D/+*^ mice (n = 8) and *Kras*^*G12D/+*^*;Cox-2*^*−/−*^ mice (n = 8). Eight mice from each group were used for quantification. Magnifications: 200x. Data are mean ± SD. *p* values are determined by the Student’s *t*-test. *p < 0.05; ***p < 0.001; ****p < 0.0001

Ablation of COX-2 was reported to suppress KRAS hyper-activity [11]. To further confirm that KRAS hyperactivation is a determinant of enhanced aerobic glycolysis under obesogenic HFD challenge, we employed the *Kras*^*LSL-G12D/+*^*;Cox-2*^*flox/flox*^*;fElas*^*CreERT*^ (hereafter called *Kras*^*G12D/+*^*;Cox-2*^*−/−*^) mice fed a HFD for 10 weeks as in Fig. 1a. As expected, COX-2 ablation led to a significant reduction in KRAS activity (Fig. 2a-b). Correspondingly, levels of HKII and LDHA were substantially reduced in the *Kras*^*G12D/+*^*;Cox-2*^*−/−*^ mice compared to *Kras*^*G12D/+*^ mice under the same HFD challenge (Fig. 2c-e). These data suggest that the HFD-promoted oncogenic KRAS hyperactivation can be inhibited by COX-2 depletion, leading to a subsequent reduction of both HKII and LDHA. Consistently, COX-2 ablation substantially reduced PanIN lesions induced by the synergistic interaction between HFD and Kras^G12D^ (Fig. 2f-g), indicating the critical importance of KRAS hyperactivation in heightening aerobic glycolysis under HFD challenge.

## Discussion

Our study reveals that obesogenic HFD cooperates with oncogenic KRAS to aggravate aerobic glycolysis. Mechanistically, chronic consumption of HFD hyper-activates mutant KRAS to promote aerobic glycolysis compared to CD, leading to rapid and wide-spread PanIN lesions. Conversely, inhibition of oncogenic KRAS hyperactivation by abrogating COX-2 prevents the HFD-enhanced aerobic glycolysis and high-grade dysplasia. Previous studies showed that oncogenic KRAS is a master regulator of aerobic glycolysis [7, 8, 10], while our study demonstrates that chronic consumption of HFD synergizes with oncogenic KRAS to drive heightened aerobic glycolysis. Thus, our data unravel a potential mechanism by which obesogenic HFD as an important environmental risk factor conspires with oncogenic KRAS to fuel aerobic glycolysis that is associated with critical pathogenic responses in the path toward PDAC.

**Fig. 3:**
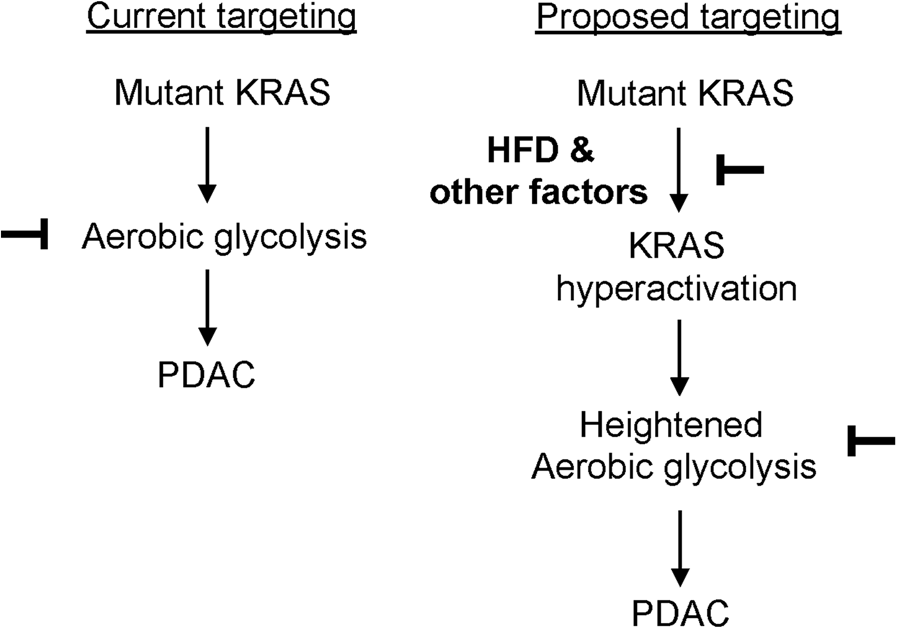
Targeting the synergistic interaction between HFD and oncogenic KRAS to inhibit aerobic glycolysis. Previous studies have shown that oncogenic KRAS drives aerobic glycolysis to facilitate PDAC development, and thus, targeting oncogenic KRAS downstream pathways responsible for aerobic glycolysis or directly inhibiting aerobic glycolysis has been proposed as therapeutic strategies to hamper PDAC development. In this study, we demonstrate that chronic consumption of obesogenic high-fat diet (HFD), but not carbohydrate-rich diet, synergizes with oncogenic KRAS to induce heightened aerobic glycolysis in PDAC development. Therefore, targeted inhibition of this synergistic cooperation would be a novel approach to inhibiting aerobic glycolysis for the prevention and treatment of PDAC

Currently, surgical resection is the only potential treatment that offers a cure for pancreatic cancer; however, only 15–20% of patients are eligible candidates for pancreatectomy. Beyond surgery, treatment options are limited to 5-fluorouracil or gemcitabine-based regimens, such as the combination of nab-paclitaxel (albumin-conjugated paclitaxel) and gemcitabine, the combination of the epidermal growth factor receptor (EGFR) inhibitor erlotinib and gemcitabine, and the FOLFIRINOX (folinic acid, fluorouracil, irinotecan, and oxaliplatin) regimen [18–20]. In addition, targeted inhibition of KRAS and its downstream effectors, such as the PI3K-AKT and mTOR (the mammalian target of rapamycin) pathway, the RAC1 signaling network, the RAL small GTPase effector signaling network, and the RAF-MEK-ERK pathway, have been actively pursued [21]. Despite these tremendous efforts, pancreatic cancer remains an intractable disease. Given the critical role of oncogenic KRAS in controlling cancer metabolism, a growing number of studies have been focusing on targeting the metabolic vulnerabilities conferred by oncogenic KRAS [7, 22–26]. Obesity, as a modifiable risk factor for PDAC, has been increasing at an alarming rate and is reaching the epidemic proportion [27]. Our studies demonstrate that obesogenic HFD enhances aerobic glycolysis and pancreatic pathogenic responses through hyperactivation of oncogenic KRAS. COX-2, a known pro-inflammatory enzyme responsible for the generation of several inflammation mediators including prostaglandin E2, has been shown to promote pancreatic cancer development [15, 28]. Importantly, out studies have shown that COX-2 ablation dramatically inhibited the synergistic interaction between HFD and mutant KRAS in enhancing aerobic glycolysis and pancreatic tumorigenesis, indicating that targeted inhibition of COX-2 might be an effective intervention strategy for the suppression of oncogenic KRAS-driven pancreatic cancer metabolism in obese patients. Thus, our studies suggest that in addition to directly targeting oncogenic KRAS and its downstream effectors and glycolytic pathways [29–32], targeting the upstream inducers of KRAS hyperactivation may constrain the heightened aerobic glycolysis for cancer prevention and therapy (Fig. 3).

In addition to the elevation of aerobic glycolysis, oncogenic KRAS also drives other metabolic alterations, such as the enhancement of nutrients uptake, fatty acids and nucleotides synthesis, glutaminolysis, and glutathione synthesis [33]. Mitochondria are metabolic hubs containing enzymes, metabolic intermediates, and energy currencies important for several key metabolic circuitries, including fatty acid β-oxidation, TCA cycle, electron transport chain, and biosynthesis of building blocks for cell proliferation [34, 35]. Therefore, mitochondria are also critically involved in cancer metabolism [34]. It is desirable to determine the alterations of the mitochondrial TCA cycle, fatty acid β-oxidation, and oxidative phosphorylation induced by HFD and oncogenic KRAS. Recent studies have also shown that oncogenic KRAS signaling activates NRF2, which is a key redox regulator important for cellular glutathione biosynthesis and plays a critical role in pancreatic cancer cell transformation, survival, proliferation, and metabolism [36, 37]. Chronic HFD consumption is known to induce oxidative stress. Thus, understanding how HFD and oncogenic KRAS alter the redox status and cellular glutathione levels should provide new insights into the design of redox-based cancer therapeutics.

Several key signaling cascades, such as the PI3K/AKT/mTOR signaling axis, the 5′-AMP-activated protein kinase (AMPK), hypoxia and HIF1α, p53, and MYC, were shown to be invovled in metabolic reprogramming of cancer cells to achieve the proliferative state. For example, AMPK, as a metabolic sensor, increases ATP levels by promoting glucose uptake [38], controls overall cellular lipid metabolism by directly phosphorylating ACC1 (acetyl-CoA carboxylase 1) and ACC2 [39], and regulates mitochondrial homeostasis by controlling mitochondrial fission, mitophagy, and biogenesis [40]. AMPK can either repress or promote tumor growth depending on the context [41, 42]. It would be of interest to investigate the roles of the aforementioned metabolic signaling cascades in the context of HFD and oncogenic KRAS.

In short, our data indicate that oncogenic KRAS requires further activation to enhance aerobic glycolysis, highlighting a pivotal role of HFD in augmenting KRAS activity to promote metabolic rewiring during pancreatic tumorigenesis. In light of our new findings, we suggest that understanding the underlying mechanisms of HFD-promoted oncogenic KRAS hyperactivation is critical for the design of therapeutic strategies for  KRAS-mediated metabolic reprogramming and malignancies.

## Abbreviations

ACC: Acetyl-CoA carboxylase
AKT: Protein kinase B
AMPK: 5′-AMP-activated protein kinase
CD: Carbohydrate-rich diet
COX-2: Cyclooxygenase-2
EGFR: Epidermal growth factor receptor
ERK: Extracellular signal-regulated kinase
FOLFIRINOX: Folinic acid, fluorouracil, irinotecan, and oxaliplatin
GEF: Guanine nucleotide exchange factor
HFD: High-fat diet
HIF-1: Hypoxia-inducible factor-1
HKII: Hexokinase II
KRAS: Kirsten rat sarcoma viral oncogene homolog
LDHA: Lactate dehydrogenase A
MAPK: Mitogen-activated protein kinase
mTOR: The mammalian target of rapamycin
MYC: Myc proto-oncogene protein
NRF2: Nuclear factor erythroid 2–related factor 2
PanIN: Pancreatic intraepithelial neoplasia
PDAC: Pancreatic ductal adenocarcinoma
PI3K: Phosphatidylinositol 3-kinase
RAC1: Ras-related C3 botulinum toxin substrate 1
ROS: Reactive oxygen species
TCA: Cycle tricarboxylic acid cycle

## References

[1] Pavlova NN, Thompson CB (2016). The emerging hallmarks of cancer metabolism. Cell Metab.

[2] Vander Heiden MG, DeBerardinis RJ (2017). Understanding the intersections between metabolism and cancer biology. Cell..

[3] DeBerardinis RJ, Thompson CB (2012). Cellular metabolism and disease: what do metabolic outliers teach us?. Cell..

[4] Cairns RA, Harris IS, Mak TW (2011). Regulation of cancer cell metabolism. Nat Rev Cancer.

[5] Liberti MV, Locasale JW (2016). The Warburg effect: how does it benefit cancer cells?. Trends Biochem Sci.

[6] Jones S, Zhang X, Parsons DW, Lin JC, Leary RJ, Angenendt P (2008). Core signaling pathways in human pancreatic cancers revealed by global genomic analyses. Science..

[7] Ying H, Kimmelman AC, Lyssiotis CA, Hua S, Chu GC, Fletcher-Sananikone E (2012). Oncogenic Kras maintains pancreatic tumors through regulation of anabolic glucose metabolism. Cell..

[8] Hu Y, Lu W, Chen G, Wang P, Chen Z, Zhou Y (2012). K-ras(G12V) transformation leads to mitochondrial dysfunction and a metabolic switch from oxidative phosphorylation to glycolysis. Cell Res.

[9] Collins MA, Bednar F, Zhang Y, Brisset JC, Galban S, Galban CJ (2012). Oncogenic Kras is required for both the initiation and maintenance of pancreatic cancer in mice. J Clin Invest.

[10] Kerr EM, Gaude E, Turrell FK, Frezza C, Martins CP (2016). Mutant Kras copy number defines metabolic reprogramming and therapeutic susceptibilities. Nature..

[11] Philip B, Roland CL, Daniluk J, Liu Y, Chatterjee D, Gomez SB (2013). A high-fat diet activates oncogenic Kras and COX2 to induce development of pancreatic ductal adenocarcinoma in mice. Gastroenterology..

[12] Ji B, Song J, Tsou L, Bi Y, Gaiser S, Mortensen R (2008). Robust acinar cell transgene expression of CreErT via BAC recombineering. Genesis..

[13] Kopp JL, von Figura G, Mayes E, Liu FF, Dubois CL, Morris JP (2012). Identification of Sox9-dependent acinar-to-ductal reprogramming as the principal mechanism for initiation of pancreatic ductal adenocarcinoma. Cancer Cell.

[14] Huang H, Daniluk J, Liu Y, Chu J, Li Z, Ji B (2014). Oncogenic K-Ras requires activation for enhanced activity. Oncogene..

[15] Daniluk J, Liu Y, Deng D, Chu J, Huang H, Gaiser S (2012). An NF-kappaB pathway-mediated positive feedback loop amplifies Ras activity to pathological levels in mice. J Clin Invest.

[16] Rodriguez-Viciana P, Tetsu O, Oda K, Okada J, Rauen K, McCormick F (2005). Cancer targets in the Ras pathway. Cold Spring Harb Symp Quant Biol.

[17] Guerra C, Collado M, Navas C, Schuhmacher AJ, Hernandez-Porras I, Canamero M (2011). Pancreatitis-induced inflammation contributes to pancreatic cancer by inhibiting oncogene-induced senescence. Cancer Cell.

[18] Moore MJ, Goldstein D, Hamm J, Figer A, Hecht JR, Gallinger S (2007). Erlotinib plus gemcitabine compared with gemcitabine alone in patients with advanced pancreatic cancer: a phase III trial of the National Cancer Institute of Canada clinical trials group. J Clin Oncol.

[19] Von Hoff DD, Ervin T, Arena FP, Chiorean EG, Infante J, Moore M (2013). Increased survival in pancreatic cancer with nab-paclitaxel plus gemcitabine. N Engl J Med.

[20] Conroy T, Desseigne F, Ychou M, Bouche O, Guimbaud R, Becouarn Y (2011). FOLFIRINOX versus gemcitabine for metastatic pancreatic cancer. N Engl J Med.

[21] Waters AM, Der CJ (2018). KRAS: The Critical Driver and Therapeutic Target for Pancreatic Cancer. Cold Spring Harb Perspect Med.

[22] Wong CC, Qian Y, Li X, Xu J, Kang W, Tong JH (2016). SLC25A22 promotes proliferation and survival of colorectal cancer cells with KRAS mutations and xenograft tumor progression in mice via intracellular synthesis of aspartate. Gastroenterology..

[23] Shah YM, Lyssiotis CA (2016). Mitochondrial amino acid metabolism provides vulnerabilities in Mutant KRAS-driven cancers. Gastroenterology.

[24] Vander Heiden MG (2011). Targeting cancer metabolism: a therapeutic window opens. Nat Rev Drug Discov.

[25] Weinberg F, Hamanaka R, Wheaton WW, Weinberg S, Joseph J, Lopez M (2010). Mitochondrial metabolism and ROS generation are essential for Kras-mediated tumorigenicity. Proc Natl Acad Sci U S A.

[26] Hosein AN, Beg MS (2018). Pancreatic cancer metabolism: molecular mechanisms and clinical applications. Curr Oncol Rep.

[27] Stevens J, Oakkar EE, Cui Z, Cai J, Truesdale KP (2015). US adults recommended for weight reduction by 1998 and 2013 obesity guidelines, NHANES 2007-2012. Obesity (Silver Spring).

[28] Kamei K, Ishikawa TO, Herschman HR (2006). Transgenic mouse for conditional, tissue-specific Cox-2 overexpression. Genesis..

[29] Le A, Cooper CR, Gouw AM, Dinavahi R, Maitra A, Deck LM (2010). Inhibition of lactate dehydrogenase a induces oxidative stress and inhibits tumor progression. Proc Natl Acad Sci U S A.

[30] Maurer T, Garrenton LS, Oh A, Pitts K, Anderson DJ, Skelton NJ (2012). Small-molecule ligands bind to a distinct pocket in Ras and inhibit SOS-mediated nucleotide exchange activity. Proc Natl Acad Sci U S A.

[31] Patra KC, Wang Q, Bhaskar PT, Miller L, Wang Z, Wheaton W (2013). Hexokinase 2 is required for tumor initiation and maintenance and its systemic deletion is therapeutic in mouse models of cancer. Cancer Cell.

[32] Ryan MB, Der CJ, Wang-Gillam A, Cox AD (2015). Targeting RAS-mutant cancers: is ERK the key?. Trends Cancer.

[33] Biancur DE, Paulo JA, Malachowska B, Quiles Del Rey M, Sousa CM, Wang X (2017). Compensatory metabolic networks in pancreatic cancers upon perturbation of glutamine metabolism. Nat Commun.

[34] Vyas S, Zaganjor E, Haigis MC (2016). Mitochondria and cancer. Cell..

[35] Cannino G, Ciscato F, Masgras I, Sanchez-Martin C, Rasola A (2018). Metabolic plasticity of tumor cell mitochondria. Front Oncol.

[36] Chio IIC, Jafarnejad SM, Ponz-Sarvise M, Park Y, Rivera K, Palm W (2016). NRF2 promotes tumor maintenance by modulating mRNA translation in pancreatic cancer. Cell..

[37] DeNicola GM, Karreth FA, Humpton TJ, Gopinathan A, Wei C, Frese K (2011). Oncogene-induced Nrf2 transcription promotes ROS detoxification and tumorigenesis. Nature..

[38] Hardie DG (2013). AMPK: a target for drugs and natural products with effects on both diabetes and cancer. Diabetes..

[39] Hardie DG, Pan DA (2002). Regulation of fatty acid synthesis and oxidation by the AMP-activated protein kinase. Biochem Soc Trans.

[40] Herzig S, Shaw RJ (2018). AMPK: guardian of metabolism and mitochondrial homeostasis. Nat Rev Mol Cell Biol.

[41] Eichner LJ, Brun SN, Herzig S, Young NP, Curtis SD, Shackelford DB (2019). Genetic analysis reveals AMPK is required to support tumor growth in murine Kras-dependent lung cancer models. Cell Metab.

[42] Faubert B, Boily G, Izreig S, Griss T, Samborska B, Dong Z (2013). AMPK is a negative regulator of the Warburg effect and suppresses tumor growth in vivo. Cell Metab.

